# Posttraumatic Stress and Perceived Interpersonal Provocation in Adolescents

**DOI:** 10.1177/08862605221104525

**Published:** 2022-05-25

**Authors:** Jonathan Marshall Saunderson, Andrew Stickley, Knut Sturidsson, Roman Koposov, Denis G. Sukhodolsky, Vladislav Ruchkin

**Affiliations:** 1 Department of Clinical Neuroscience, Division of Psychology, Karolinska Institute, 27106 Stockholm, Sweden; 2 Department of Preventive Intervention for Psychiatric Disorders, National Institute of Mental Health, National Center of Neurology and Psychiatry, Kodaira, Tokyo, Japan; 3 Stockholm Center for Health and Social Change (SCOHOST), Södertörn University, Huddinge, Sweden; 4 Regional Centre for Child and Youth Mental Health and Child Welfare, Faculty of Health Sciences, UiT The Arctic University of Norway, 8016 Tromsø, Norway; 5 Sechenov First Moscow State Medical University, Moscow, Russia; 6 Child Study Center, Yale University Medical School, 12228 New Haven, CT, USA; 7 Department of Child and Adolescent Psychiatry, Division of Neuroscience, Uppsala University, 8097 Uppsala, Sweden; 8 Säter Forensic Psychiatric Clinic, Säter, Sweden

**Keywords:** adolescents, posttraumatic stress, aggression, gender, interpersonal provocation

## Abstract

**Objective:** To examine the impact of posttraumatic stress on the choice of responses to and attribution of intentionality in peer provocation in adolescent boys and girls. **Methods:** A sample of 2678 adolescents from Northern Russia, aged 13–17 years (59.3% female; 95.7% ethnic Russian) completed self-reports on posttraumatic stress and rated hypothetical peer provocation scenarios that teenagers can encounter in their daily lives. **Results:** Adolescents with clinically significant levels of posttraumatic stress symptoms (*n*=184 (6.8%)) reported a different pattern of reactions to peer provocation as compared to all other adolescents. Boys and girls with high levels of posttraumatic symptoms reported that they would be less likely to discuss conflict situations and more likely to react with physical aggression. Compared to their male counterparts, girls with high levels of posttraumatic stress symptoms were more likely to endorse hostile intentions, avoid provocations, and were less likely to endorse verbally aggressive responses. In provocation scenarios that involved physical aggression, girls with high levels of posttraumatic stress symptoms were less likely to endorse verbal aggressive responses and more likely to endorse physically aggressive responses than girls without clinically significant levels of posttraumatic symptoms. Girls with high levels of posttraumatic stress symptoms were also more likely to avoid socially aggressive situations than non-traumatized girls, whereas boys had an opposite pattern. **Conclusions:** High levels of posttraumatic stress symptoms may play a significant role in the endorsement of aggressive reactions in conflicts with peers and patterns of reactions may be gender-specific. A history of posttraumatic stress should be carefully evaluated in children and adolescents seeking treatment for aggressive behavior.

## Introduction

Stress is a natural physiological and behavioral response to potentially dangerous or life-threatening events (Yaribeygi et al., 2017). It is induced by environmental forces and is manifested by reactions at various physiological, behavioral, and social levels and is thus defined by the functional relationships between antecedent events and their adverse consequences (Kendall & Hollon, 1979). It serves in allowing an individual to adjust perception and interpretation of potentially harmful stimuli and to react accordingly (Mariotti, 2015). Individual perceptions and experiences of traumatic events are processed based upon prior and current knowledge and context, which are then interpreted to form specific behavioral responses (Frith, 2008). Previous experiences of severe psychological/traumatic stress may lead to profound changes in the way people react to situations and events (Amstadter & Vernon, 2008) and may have detrimental effects on an individual’s behavioral style, as well as his/her physical and mental health (Kendall & Hollon, 1979).

### Psychological trauma and the perception of interpersonal provocation

In cases of severe psychological trauma, an individual may develop posttraumatic stress disorder (PTSD), which among other symptoms is characterized by negative alterations in cognition and mood (e.g., exaggerated negative beliefs or expectations about others; persistent negative emotional states, such as fear and anger), as well as by marked alterations in arousal and reactivity (e.g., irritable behavior and angry outbursts with little or no provocation, typically expressed as verbal or physical aggression) (American Psychiatric Association, 2013). These adverse changes in individual cognitive-emotional responses may become especially apparent in situations of interpersonal provocation, that pose a potential threat, including interpretation of the situation, specific cognitive attributions that arise and resulting conflict resolution strategies (Couette et al., 2020). In such situations, an individual may perceive neutral cues surrounding an event as hostile or life-threatening, which may in turn lead to adverse reactions, even in the absence of any danger and to persistent trauma-related reactivity (Badour & Feldner, 2013).

Perceived interpersonal provocation is a common antecedent of aggression and research has shown that an individual’s perception of being exposed to provocation reduces self-control and increases the likelihood and severity of aggression (Denson et al., 2011). Aggressive behavioral responses are hence influenced by an individual’s tendency to interpret the intent of others as hostile, even when social context cues may be ambiguous (Milich & Dodge, 1984), and this may be further amplified by anger regulation deficits, such as those seen in individuals with posttraumatic stress (PTS) (e.g., Chemtob et al., 1997). Research suggests that situations of acute stress may further negatively impact cognitive and emotional regulation, and increase the likelihood that an individual will appraise neutral stimuli as harmful and react in a maladaptive way with negative emotions (Zhan et al., 2017). Hence, people with high levels of PTS may be particularly vulnerable when exposed to acute stress, such as in situations of interpersonal provocation, which pose an additional challenge to their cognitive and emotional regulation strategies, already impacted by previous traumatic experiences, hence making them more prone to appraise situations negatively.

### Reactions to interpersonal provocation in adolescents

Previous research suggests that there are three general reactions to interpersonal provocation, including withdrawal, a prosocial reaction and aggression (e.g., Lindeman et al., 1997). Accordingly, Shulman et al. (2006) reported three distinctive conflict resolution patterns in adolescents, including a downplaying/avoiding pattern characterized by a tendency to minimize conflict, an integrative pattern with an inclination to negotiate differences, and a conflictive pattern with a confrontational interaction style. Fernet et al. (2016) similarly proposed three main patterns of conflict resolution strategies in youth, including avoiding conflict or its resolution; negotiating expectations and individual needs; and imposing personal needs and rules through the use of violence. Considering the possible impact of traumatic experiences on social and emotional processing, it seems reasonable to speculate that adolescents with high levels of PTS symptoms may differ in their responses to peer provocation from other adolescents in terms of more aggressive responses and a decreased tendency to negotiate conflict.

### Gender and interpersonal provocation

It has been further suggested that reactions to interpersonal provocation may differ by gender. In particular, findings suggest that boys tend to be more verbally and physically aggressive than girls, and that girls are more likely to use social rather than physically aggressive strategies to harm a target (see Heilbron and Prinstein (2008) for a review). Adolescent girls also tend to score higher in communication skills (Black, 2000) and to react in a more prosocial/negotiating way than boys (Eisenberg et al., 1991), whereas both boys (Black, 2000; Lindeman et al., 1997) and adult males (Brahnam et al., 2005) may be more prone to withdrawal/avoidant behavior. The latter may however change as the type of provocation changes, since when already involved in conflict, men are more likely to use aggression and control strategies, compared to women (e.g., Feldman & Gowen, 1998).

### Gender and reaction to stress

At the same time, there are well-documented gender differences in the reaction to psychological stress. Research suggests that in response to stress, women, as compared to men, are more likely to experience heightened levels of negative emotional expression, such as fear, anxiety, and helplessness (Chaplin et al., 2008; Lilly et al., 2009) and report a higher prevalence of mental health problems (Axinn et al., 2013), while men tend to experience higher levels of subjective anger and less fear (Chaplin et al., 2008). One can hence hypothesize that severe PTS not only impacts on individual conflict resolution strategies, but these strategies may be expressed in a gender-specific manner and may vary depending on the type of provocation.

### Cross-cultural perspective*s* on trauma and aggression

Hence, there is substantial empirical evidence concerning the associations between PTS and irritability and aggressive behavior. However, as yet, research about the effects of PTS on perceived interpersonal provocation, especially in different cross-cultural contexts, has been limited. Similar to US youth, previous studies with Russian adolescents suggest that PTS may have an important impact on anger, anger rumination, and aggression (Isaksson et al., 2020) and that there may be significant gender differences in aggression and conflict resolution patterns, with boys scoring higher on physical aggression (Butovskaya et al., 2007), especially in the presence of PTS symptoms (Isaksson et al., 2020). There are also consistent similarities in findings on anger and the associations of anger to aggression in Russian adolescents and young adults (Kassinove et al., 1997). Likewise, among Russian adolescents aggressive behavior was found to be significantly associated with higher levels of anger and stronger beliefs that physical aggression is an appropriate course of action in conflicts (Sukhodolsky & Ruchkin, 2004). However, some authors have suggested that there may be cross-cultural differences in beliefs about aggressive behavior among Russian and American adolescents (Finckenauer, 2001) and considering the current climate in Russia, it is possible that these youth may historically perceive PTS and aggression differently, and therefore represent a unique population to be considered (Fitzpatrick, 2004; Kelly, 2007). Hence, the current study extended previous social-cognitive research on aggression and interpersonal provocation in youth by using a large general population sample of adolescents from a cultural background outside of North America and Western Europe to investigate the association between clinical levels of PTS symptoms and reactions to peer provocation.

The purpose of this study was to therefore examine the impact of PTS on the choice of responses to and attribution of intentionality in peer provocation in adolescent boys and girls. It was hypothesized that the relationship between clinical levels of PTS and an adolescent’s reaction to peer provocation may vary among boys and girls and that the type of reaction may differ further depending on the type of provocation.

## Method

### Participants

This study was approved by institutional review committees at the Northern State Medical University (Arkhangelsk, Russia). The study was conducted in Arkhangelsk, a large city in the northwestern part of European Russia. The socio-economic status of the population is rated low to average in comparison to all of Russia, and inter-individual differences in socio-economic status such as age and gender are marginal.

According to census data (Russian Federal State Statistics Service, 2011), the population of Arkhangelsk is slightly under 349,000, with approximately 30,000 being aged 12–17 years old. Permission to conduct this study in selected schools was obtained from the Arkhangelsk city administration and the study was conducted in collaboration with the local schools’ administration. A randomized selection procedure was used to obtain a representative sample with different schools and classes as units of randomization. The study involved all the main districts of the city and the number of potential participants from each district was calculated in proportion to the total number of residents of the relevant age in the district. The randomized selection procedure occurred in 2 stages. In stage one, 14 schools were randomly selected from 71 eligible public schools, all of which agreed to participate and were included in the study, yielding 210 classes in grades 6 to 11. In stage two, data were collected from students in 70 randomly selected classes, resulting in a sample of 2892 eligible students. Students with incomplete reports were excluded, rendering a final sample of 2678 student participants. Youths from the excluded group were more likely to be male (62.1%, Chi-square = 30.63, *p* < .001), but otherwise did not differ from the participants on any demographic variable or any other variable of interest. Participants ranged in age from 13 to 17 years old. Females comprised 59.3% of the sample (*n* = 1589), and 95.7% were predominantly of Russian origin (*n*=2564) followed by a small proportion of Ukrainians, Byelorussians, and other ethnicities, an accurate reflection of the local residential population (57% female; 95.6% Russian ethnicity) and of the local public school population (Russian Federal States Statistics Service, 2011). Most of the participants (75.6%) came from two-parent families, whereas 24.4% had divorced, separated, or widowed parents. According to the students’ reports, 93.0% of their fathers and 94.4% of their mothers had completed, at a minimum, the equivalent of a high school education or higher.

### Procedure

Parents of students were initially informed of the survey and were offered the opportunity to decline participation. Before the survey was administered, students were read a complete assent form outlining their participation and confidentiality and were asked to sign it to indicate assent. Students also had the option to decline to participate at the time of the survey’s administration (parent and student refusals were less than 1%). Students completed the survey questionnaire in one class period during a normal school day. The questionnaires were administered in Russian.

### Measures

The *Child Self-Report Post-Traumatic Stress Reaction Index* (CPTS-RI) is a 20-item scale designed to assess PTS symptoms in school-aged children and adolescents after exposure to a broad range of traumatic events (Pynoos et al., 1987, 1993). The frequency of symptoms is assessed on a Likert-type five-point rating scale ranging from “never” (0) to “most of the time” (4), where the total score can range from 0 to 80. The scale is internationally recognized, continuously updated to the current DSM-criteria for PTSD and has well-established cross-cultural clinical cut-offs according to the raw score, where the degree of reaction is categorized as doubtful (score<12), mild (score=12–24), moderate (score=25–39), severe (score=40–59), or very severe (score>60). Higher clinical scores on this instrument correlate closely with the DSM diagnosis of PTSD (Pynoos et al., 1993), including in Russian youth (Ruchkin et al., 2002). The translation of the scale into Russian followed established guidelines, including the appropriate use of independent back-translations (Sartorius & Kuyken, 1994). Cronbach’s alpha for the scale was .86. For the present study, all participants were divided into two groups based on their CPTS-RI score. Those with a score of 40 and above were categorized as having a clinical level of PTS, while those with a score of 39 and below were categorized as having a subclinical level of PTS.

In addition to the 20 items assessing the frequency of PTS symptoms, we complemented the questionnaire with three additional items inquiring about the subjective degree of functional impairment associated with the symptoms in three areas (Think about the questions you just answered. Did any of these feelings cause problems for you… 1) at school, 2) with friends and 3) at home), corresponding to the functional impairment criteria in DSM-5 (American Psychiatric Association, 2013). Each item was scored on a 5-point scale, varying from, “Not at all” (1) to “A lot” (5), hence producing a total functional impairment score, potentially ranging from 3 to 15. Cronbach’s alpha for the scale was .82.

A proxy for s*ocio-economic status* (SES) was created using students' reports on single-family status (1/0), lower level parental education (incomplete college education or lower, 1/0), and parental employment status (full time (0), part-time (1), and unemployed (2)). A continuous variable was subsequently created where higher scores represent lower SES.

The *Reaction to Peer Provocation (RPP) questionnaire* was created by the authors (DS and VR) for the purposes of the present study and consisted of 12 short scenarios that describe hypothetical peer provocations that can be encountered by teenagers in schools or in the community. The provocations included four scenarios that relate to social aggression (e.g., “you have learned that your classmate is spreading nasty rumors about you”), four scenarios depicting verbal provocation (e.g., “a classmate made a joke about your appearance in front of your friends”), and four scenarios of physical aggression (“during an argument, another kid pushed you with both hands,” or “a classmate kicked you from the back so that you almost fell”). Each scenario was followed by two questions: “how would you react in this situation?” (i.e., the type of reaction) and “how would you describe the behavior of this person?” (i.e., the perceived hostile intention). Respondents were asked to select one of four response categories to indicate their most likely reaction in each scenario: “I would ignore this situation” (=avoidance), “I would calmly discuss and solve this problem” (=negotiation), “I would raise my voice, curse at the person, or call him/her a name” (=verbal aggression), and “I would push, shove or kick another person” (=physical aggression). Separate scores were calculated for each of the four types of reaction (avoidance, negotiation, verbal aggression, and physical aggression), each potentially ranging from 0 to 12. In addition, subscores were summed for each type of reaction (avoidance, negotiation, verbal aggression, physical aggression) in three separate contexts (social aggression, verbal aggression, physical aggression), each potentially ranging from 0 to 4, with higher scores indicating a greater level of each type of reaction. The question on the perceived hostile intention of the provocateur in each scenario was answered by selecting one of four responses: “Definitely on purpose (=3),” “Possibly on purpose (=2),” “Possibly an accident (=1),” “Definitely an accident (=0).” A total score for perceived hostile intention was calculated as a total for all 12 scenarios, with a possible range from 0 to 36, with higher scores indicating a greater hostile attribution bias.

### Statistical analyses

Data were analyzed using the Statistical Package for the Social Sciences (SPSS-25.0). Chi-square and independent sample t-tests were used for univariate comparisons of demographic characteristics and of dependent variables across gender.

General linear models (GLM) multivariate analysis of covariance (MANCOVA) was then used to determine main and interaction effects across the fixed factors of PTS group (clinical level vs. subclinical level) and gender (boys=1, girls=0) while adjusting for the age and SES covariates. Two separate MANCOVA analyses were conducted for the general reaction to interpersonal provocation variables (avoidance, negotiation, verbal aggression, physical aggression, and the perceived hostile intention of the action), as well as a more detailed analysis of reactions to specific types of conflict (social provocation, verbal provocation, and physical provocation). Hence, we used a 2 (PTS group) X 2 (gender) design for each cluster of variables.

The unique contribution of each of the two fixed factors, the one interaction term, and the two covariates was assessed through follow-up between-subject tests and unstandardized parameter estimates derived from the MANCOVA. Results are presented as means (M) and standard deviations (SD), and for individual outcomes, as partial eta squared (η2), a common metric of effect size that represents the unique amount of variance explained by each predictor variable, in which the effects of other independent variables and interactions are partialled out. Cohen (1988) provided points of reference to define small (η2 = 0.01), medium (η2 = 0.06), and large (η2 = 0.14) effects.

## Results

### Prevalence of posttraumatic stress and associated functional impairment by gender

A total of 184 (6.8%) adolescents reported levels of PTS that were considered as severe or very severe, according to the established cut-offs. The t-test comparison by gender showed that girls generally reported higher levels of PTS than boys (M(SD) = 21.40 (11.49)) versus 17.67 (11.16), t=8.40, *p*<.001. The Univariate ANOVA, comparing the levels of subjective functional impairment associated with PTS by gender and controlling for the SES proxy and age, showed significant effects for the PTS group (F (1, 2679) = 262.84, *p*<.000, η2 = .086) and for gender (F (1, 2679) = 16.04, *p*<.000, η2 = .006), while the interaction effect for the PTS group by gender was non-significant (F (1, 2679) = .26, ns, η2 = .000), indicating that girls generally reported higher levels of functional impairment than boys, that those with clinical PTS reported higher levels of functional impairment than those with subclinical PTS, and that there was no gender-specific functional impairment in relation to PTS level (M(SD) = 6.38 (3.36) vs. 3.20 (2.67) for girls with clinical vs. subclinical levels of PTS, and 5.64 (3.68) vs. 2.27 (2.19) for boys, respectively). The effect for age was only weakly significant (F (1, 2679) = 5.50, *p*<.05, η2 = .002), while the effect for the SES proxy was not significant (F (1, 2679) = .42, ns, η2 = .000).

### Generalized Linear Modeling

When assessing differences in the general reaction to peer provocation (avoidance, negotiation, verbal aggression, and physical aggression) and in perceived hostile intentions by PTS group (see Table 1 for descriptive statistics (M (SD)) by gender and Table 2 for the main effects and the tests of between-subjects effects), the main effect for PTS level was significant, suggesting that the potential reaction to provocation differed between those with clinical and subclinical PTS. The main effect for gender was significant, showing a difference in the reported reaction to peer provocation between boys and girls. The main effect for age was not significant, indicating that there was no difference in the reported reactions to peer provocation by age. The main effect for SES was also not significant. Finally, the interaction effect for PTS level by gender was significant, which indicates that the reactions to peer provocation in relation to PTS were gender-specific. Table 2 presents effect sizes for each dependent variable (avoidance, negotiation, verbal aggression, and physical aggression, as well as for the perceived hostile intention of the action). The results suggest that the significant main effect for PTS level was primarily related to a lower willingness to negotiate and a greater tendency to respond with physical aggression in potential conflict situations in those with clinical PTS. As concerns gender differences (the main effect for gender), girls were more likely to endorse negotiation in a conflict situation than were boys. Girls as a group also tended to report using more verbal aggression and less physical aggression, as well as to ascribe hostile intentions less often than boys. As concerns the gender-specific differences in relation to PTS (the interaction effect for PTS by gender), girls with clinical PTS reported that they would more likely avoid conflict and would less likely react with verbal aggression to a provocation than girls with subclinical PTS (opposite to the pattern for boys), even though they more often ascribed hostile intentions in such situations (opposite to the pattern seen in boys).

**Table 1.** Reaction to hypothetical peer provocation situations (M (SD)) by PTS level in boys (B) and girls (G).

Reaction to Peer Provocation		Clinical PTS	Subclinical PTS	Total Group
Avoidance	B	1.91 (1.88)	2.42 (2.32)	2.40 (2.30)
	G	2.53 (2.15)	2.18 (2.07)	2.21 (2.08)
Negotiation	B	2.64 (2.38)	3.39 (2.35)	3.35 (2.35)
	G	3.58 (2.25)	4.14 (2.52)	4.09 (2.51)
Verbal aggression	B	4.02 (2.53)	3.19 (2.18)	3.23 (2.20)
	G	4.13 (2.20)	4.30 (2.41)	4.29 (2.39)
Physical aggression	B	3.38 (3.05)	2.77 (2.47)	2.80 (2.50)
	G	1.55 (1.55)	1.27 (1.49)	1.29 (1.50)
Attributed hostile intentions	B	33.45 (8.19)	35.13 (7.06)	35.05 (7.13)
	G	36.05 (7.31)	34.76 (5.87)	34.87 (6.01)

*Note.* M(SD) – mean (standard deviation); PTS – posttraumatic stress.

**Table 2.** Effect sizes for the main factors (Wilks’ lambda; F (df); η^2^; *p*) and for each dependent variable (reaction to hypothetical peer provocation) (η^2^, *p*).

	Effect	Avoidance	Negotiation	Verbal Aggression	Physical Aggression	Attributed Hostile Intention
Age	.996; 2.03 (4, 2678); .004; ns	.001, ns	001, ns	.000, ns	.000, ns	.002, <.05
SES	.998; .911 (4, 2678); .002; ns	.000, ns	.001, ns	.000, ns	.000, ns	.001, ns
Gender	956; 24.72 (4, 2678); .044; <.001	.000, ns	.006, <.001	.004, <.01	.037, <.001	.002, <.05
PTS level	.995; 2.96 (4, 2678); .005; <.05	.000, ns	.004, <.01	.001, ns	.003, <.01	.000, ns
PTS level by gender	.989; 5.98 (4, 2678); .011; <.001	.002, <.05	.000, ns	.002, <.05	.000, ns	.003, <.01

*Note.* SES– socio-economic status; PTS- posttraumatic stress; ns –non-significant.

When assessing the differences in potential reactions to specific types of peer provocation (social aggression, verbal aggression and physical aggression) by PTS group (see Table 3 for descriptive statistics (M (SD)) by gender and Table 4 for the tests of between-subjects effects), the main effect for PTS level was significant (Wilks’ lambda=.991; F (12, 2660) = 2.09, *p*<.05, η2 = .009), suggesting that potential conflict resolution strategies in specific contexts differed between those with clinical and subclinical PTS. The main effect for gender was also significant (Wilks’ lambda=.947; F (12, 2660) = 12.33, *p*<.000, η2 = .053), indicating differences in described conflict resolution strategies between boys and girls. The main effect for age was also significant (Wilks’ lambda=.988; F (12, 2660) = 2.72, *p*<.01, η2 = .012), demonstrating differences in the chosen conflict resolution strategies by age. The main effect for SES was not significant (Wilks’ lambda=.997; F (12, 2660) = .644, ns, η2 = .003). Finally, the interaction effect for PTS level by gender was significant (Wilks’ lambda=.988; F (12, 2660) = 2.61, *p*<.01, η2 = .012), which indicates that some of the patterns of conflict resolution strategies chosen for the specific types of peer provocation in relation to PTS were gender-specific.

**Table 3.** Reaction to hypothetical peer provocation (M (SD)) by the type of provocation, PTS level in boys (B) and girls (G).

Type of Provocation	Reaction to Provocation		Clinical PTS	Subclinical PTS	Total Group
Social	Avoidance	B	.91 (1.02)	1.12 (.98)	1.11 (.98)
Aggression		G	1.14 (1.05)	.92 (.91)	.94 (.93)
	Negotiation	B	1.07 (1.05)	1.53 (1.11)	1.51 (1.11)
		G	1.72 (1.19)	1.95 (1.18)	1.93 (1.18)
	Verbal aggression	B	1.18 (1.07)	.73 (.90)	.75 (.91)
		G	1.01 (1.12)	.96 (1.06)	.96 (1.07)
	Physical aggression	B	.84 (1.01)	.57 (.85)	.59 (.86)
		G	.13 (.43)	.14 (.43)	.14 (.44)
Verbal	Avoidance	B	.65 (.87)	.77 (1.05)	.76 (1.04)
Aggression		G	.78 (1.02)	.75 (.97)	.75 (.98)
	Negotiation	B	.93 (1.07)	1.12 (1.08)	1.11 (1.08)
		G	1.24 (1.15)	1.39 (1.13)	1.38 (1.13)
	Verbal aggression	B	1.64 (1.27)	1.45 (1.14)	1.46 (1.14)
		G	1.76 (1.10)	1.68 (1.19)	1.69 (1.18)
	Physical aggression	B	.78 (1.12)	.61 (.99)	.62 (1.00)
		G	.19 (.57)	.14 (.47)	.15 (.48)
Physical	Avoidance	B	.35 (.55)	.55 (.87)	.54 (.85)
Aggression		G	.64 (.78)	.51 (.78)	.52 (.78)
	Negotiation	B	.64 (.99)	.76 (.93)	.75 (.94)
		G	.66 (84)	.80 (.98)	.79 (.97)
	Verbal aggression	B	1.20 (1.15)	1.04 (.95)	1.05 (.96)
		G	1.43 (1.07)	1.66 (1.13)	1.64 (1.12)
	Physical aggression	B	1.76 (1.41)	1.62 (1.18)	1.62 (1.19)
		G	1.25 (.97)	1.00 (1.04)	1.02 (1.04)

*Note.* M(SD) – mean (standard deviation); PTS – posttraumatic stress.

**Table 4.** Effect sizes for each dependent variable (reaction to hypothetical peer provocation) by the type of provocation (η^2^, *p*).

Type of Provocation	Reaction to Provocation	Age	SES	Gender	PTS Level	PTS Level by Gender
Social	Avoidance	.000, ns	.000, ns	.000, ns	.000, ns	.003, <.01
Aggression	Negotiation	.001, ns	.000, ns	.011, <.001	.005, <.001	.001, ns
	Verbal aggression	.001, ns	.000, ns	.000, ns	.003, <.01	.002, <.05
	Physical aggression	.000, ns	.000, ns	.041, <.05	.002, <.05	.002, <.01
Verbal	Avoidance	.000, ns	.000, ns	.000, ns	.000, ns	.000, ns
Aggression	Negotiation	.001, ns	.001, ns	.003, <.01	.001, ns	.000, ns
	Verbal aggression	.000, ns	.001, ns	.001, ns	.001, ns	.000, ns
	Physical aggression	.002, <.05	.000, ns	.027, <.001	.001, ns	.000, ns
Physical	Avoidance	.001, ns	.000, ns	.001, ns	.000, ns	.002, <.05
Aggression	Negotiation	.000, ns	.001, ns	.000, ns	.001, ns	.000, ns
	Verbal aggression	.001, ns	.000, ns	.008, <.001	.000, ns	.002, <.05
	Physical aggression	.001, ns	.000, ns	.014, <.001	.002, <.05	.000, ns

*Note.* SES - socio-economic status; PTS – posttraumatic stress; ns –non-significant.

Table 4 presents effect sizes for each dependent variable (avoidance, negotiation, verbal aggression, and physical aggression) by the type of conflict situation (social aggression, verbal aggression, and physical aggression). The results suggest that the significant main effect for PTS in hypothetical situations of social aggression was related to a reduced willingness to negotiate the conflict and higher levels of reported verbal and physical aggression in those with clinical PTS. In hypothetical situations of verbal aggression, there was no difference between those with clinical and subclinical PTS in the type of reaction to conflict. In hypothetical situations of physical aggression, those with clinical PTS generally described reacting with more physical aggression than other youth. As concerns gender differences (the main effect for gender), girls indicated a greater propensity to negotiate social and verbal aggression conflict situations than boys. Girls as a group also indicated that they would use less physical aggression in all types of peer provocation situations, than boys. Finally, girls more often indicated that they would use verbal aggression in hypothetical situations of physical aggression than boys. As concerns, gender-specific differences in relation to PTS (the interaction effect for PTS by gender), in hypothetical situations of social aggression girls with clinical PTS indicated that they were more likely to avoid conflict, while boys with clinical PTS reported that they would more likely use verbal or physical aggression. In hypothetical situations of physical aggression, girls with clinical PTS stated that they would more likely avoid conflict and less likely respond with verbal aggression (opposite to the pattern seen in boys).

## Discussion

In this study we sought to investigate the relationship between PTS and the reaction of adolescents to hypothetical peer provocation scenarios, and to examine whether the association would be gender specific. We hypothesized that the relationship between clinical PTS and the reaction to conflict may be different among boys and girls, and that the type of reaction may differ further depending on the type of provocation. Our findings were consistent with previous research (Margolin & Vickerman, 2007) and suggested that adolescents with a clinical level of PTS may react differently in situations of interpersonal provocation, as compared to those with subclinical PTS, and that the relationship between PTS and the reaction to provocation may be influenced by gender and by the type of interpersonal provocation.

Girls reported higher levels of clinical PTS and functional impairment due to PTS symptoms than boys. These gender differences are in line with previous research suggesting that while males are more likely to be exposed to diverse traumatic events than females (e.g., Breslau et al., 1991), females exposed to trauma are more likely than males to report PTS symptoms (e.g., Singer et al., 1995). In addition, girls generally tend to respond more emotionally to undesirable life events (Kessler & McLeod, 1984), and it has been suggested that from adolescence onwards girls have a greater risk of developing PTSD compared to boys (Alisic et al., 2014; Garza & Jovanovic, 2017)

When comparing the type of reaction to hypothetical peer provocation by gender, girls were more likely to negotiate a situation or to react with verbal aggression, whereas boys were less likely to avoid conflict, and more likely to react with physical aggression and to ascribe hostile intentions in a conflict. It has been suggested previously that gender differences with regard to aggressive behavior may be culturally instilled in women, discouraging aggressive or self-serving behavior and favoring negotiation (Holliday et al., 2015). The tendency for boys to be more physically aggressive than girls may also be due to gender differences in information processing and responses. Boys for example, are more likely to externalize negative affect and respond with anger/aggression, while girls are more likely to internalize their responses (Chen et al., 2012). Previous studies have shown that women tend to be more emotion-focused, defensive, and palliative in their coping responses, whereas men tend to be more aggressive (Olff, 2017). A previous study with Russian adolescents (Butovskaya et al., 2007) has similarly attested to significant gender differences in aggression and conflict resolution patterns, with boys scoring higher on physical aggression, and girls on indirect aggression. Girls were socially more skillful than boys in the use of peaceful means of conflict resolution. Verbal aggression was apparently more condemned in boys than in girls, while in girls verbal aggression was positively correlated with popularity (Butovskaya et al., 2007).

The results of this study further suggested significant differences in reactions to peer provocation between traumatized and non-traumatized youths, where adolescents with PTS were less likely to avoid conflict and negotiate conflict situations and more likely to react with physical aggression. This reaction pattern is often seen in traumatized individuals and suggests that some aspects of the stress reaction may work maladaptively to further perpetuate violent behavior. Several theoretical explanations have been offered to explain the link between PTS and aggressive behavior. An individual with PTS may perceive a noxious stimulus as too intense and a neutral stimulus as harmful, which may potentially lead to maladaptive coping strategies (Mariotti, 2015). PTS may also impact on behavior and emotion regulation, and the ability to identify, evaluate, and modify the experience and expression of affect (Gratz & Roemer, 2004), especially surrounding a traumatic event (Tull et al., 2007). It has been further suggested that negative cognitions and affect in PTS may be connected through associative networks with anger-related feelings, thoughts, memories, and aggressive inclinations (Taft et al., 2007). In addition, PTS may affect the way in which information is processed and undermine an individual’s ability to engage in self-protective behavior by diminishing his/her cognitive capacity to adequately identify risk and to exit hazardous situations (Orcutt et al., 2002), or by facilitating the use of aggression as a socially acceptable response in the context of maladaptive social goals (Shahinfar et al., 2001). Some theories have thus suggested that traumatized individuals may develop a tendency toward a specific cognitive bias related to the (mis)perception of threat in the context of ambiguity, which in turn leads to a threat-anger program for action and facilitates aggression (Novaco & Chemtob, 1998). Whiting and Bryant (2007) for example, found a strong association between maladaptive appraisals and post-traumatic anger. Finally, it is also possible that the effects of PTS might result in certain physiological changes that lead to traumatic experiences becoming “addictive,” thus facilitating involvement in further violent and aggressive behavior (e.g., Haapasalo & Pokela, 1999).

As concerns gender differences in traumatized individuals in response to hypothetical peer provocation, girls with clinical PTS reported that they would more likely avoid conflict and less likely react with verbal aggression, whereas boys with PTS indicated that they would less likely avoid conflict and more likely become verbally aggressive. These findings are consistent with the role that gender plays in the expression of aggression in relation to PTS (Isaksson et al., 2020). Boys and girls tend to differ not only in the magnitude of PTS, but also in the types of behavioral outcomes of trauma that may subsequently develop. Specifically, while girls tend to react with more internalizing behaviors, boys are at increased risk for developing externalizing problems (Gorman-Smith & Tolan, 1998; Miller et al., 1999). Boys also had a greater tendency to ascribe hostile intentions in a conflict situation than girls, but within each gender, boys with clinical PTS were less prone to ascribe hostile intentions than boys with subclinical PTS, whereas girls with clinical PTS were more prone to do so than their peers with lower levels of PTS. Studies have found that hostile attribution biases are associated with reactive aggressive behavior. Research has identified a number of environmental factors influencing the development of a more hostile attributional style which is enduring and behavior mediating, including a history of physical abuse, modeling of hostile attributions by adults and peers, and being reared in a culture that values self-defense and retaliation (Denson et al., 2011). In relation to the above, PTS may also be a relevant explanation for a greater hostile attributional style in girls compared to boys, as it strongly influences cognitive-emotional processes and seems more likely to lead to internalization of negative affect in trauma processing in girls. Although the specific reasons for our observed results are unclear, it is possible that the effects of PTS might interact with specific environmental factors to determine the specific expression of hostile intentions. It has been demonstrated, for example, that PTS significantly predicts physical aggression over time, and that hostile attributions may partially mediate this association, suggesting the potential utility of targeting hostile cognitions in therapy for anger and aggression (Van Voorhees et al., 2016).

This study had a number of strengths, such as being able to use data from a large sample of adolescents while also using various measures that allowed us to evaluate perceived PTS and reactions to peer provocation. However, it also had several limitations. First, although the study used a two-stage randomization procedure in order to accurately reflect the local school population, schools and classrooms were not weighted to reflect the sampling design, which might have affected the results we obtained. Second, the assessment of PTS symptoms was based on self-reports, which may have been subject to reporting bias. Specifically, despite the fact that 184 adolescents were found to have severe or very severe PTS symptoms and had greater levels of functional impairment associated with PTS, these individuals were still able to maintain an adequate level of daily functioning and to attend school. This suggests that it might have also been beneficial to assess the levels of PTS through parent-teacher evaluations or structured screening/clinical interviews. Third, the RPP questionnaire, created by the study authors, describes a set of hypothetical scenarios, and the chosen response alternatives may differ from the actual behaviors that the assessed individuals exhibit in situations of real peer provocation. In addition, psychometric data for the RPP questionnaire (and for the measure of subjective functional impairment) were not available for publication because the instruments were not validated prior to their use in the study. This is a critical issue, given how central the measures are to this study, and limits the validity and reliability of the findings. Fourth, students with incomplete reports (*n*=214) were excluded from the study, which could have impacted the results, especially considering the higher number of excluded males, as compared to females. Fifth, the present study was limited to adolescents residing in Arkhangelsk, Russia with a large majority of them (95.7%) being of predominantly Russian origin. Although similar trends in adolescents’ reactions to peer provocation and PTS/gender relationships can be seen internationally, findings may differ in varying demographic groups. Hence, future research should be conducted cross-culturally to establish the generalizability of our findings. Sixth, while statistically significant, the effect sizes that represent the unique amount of variance explained by each predictor variable were small (Cohen, 1988) and hence our findings should be interpreted with caution. Finally, as this study had a cross-sectional design, causality cannot be proved.

Clinical levels of PTS seem to be important in the expression of aggression and gender-specific reactions to peer provocation. Results indicate that some differences in reactions to peer provocation in the wake of traumatic exposure may be attributable to gender-specific mechanisms (Ashley & Swick, 2019) and suggest that PTS may be correlated with more aggressive behavioral tendencies (Zhan et al., 2017), as well as with hostile attributional styles (Tull et al., 2007). Our findings may help explain the heterogeneity of the gender-related differences in conflict situations in connection with PTS (Chen et al., 2012) and facilitate the development of gender-specific intervention strategies.

In conclusion, this study provides a description of hypothetical reactions to interpersonal provocation in relation to PTS in a large, representative sample of adolescents from Northern Russia. To our knowledge, no previous study has addressed these research issues in Russian youths and the study aimed at expanding the existing knowledge base regarding responses to interpersonal violence while providing a deeper understanding of the mechanisms behind interpersonal violence in its many iterations. Although the study sample was reflective of the local population in that it was largely mono-ethnic Russian, the results were nevertheless in line with those from other, largely North American populations, indicating that our findings may be potentially generalizable to other cultures. At the same time, even though interpersonal provocations are common triggers of anger and even aggressive behavior in adolescents around the world, social perception of provocations as well as normative and abnormal reactions to provocation can differ in different countries (Shiraev & Levy, 2020). Although this study was not designed as a cross-cultural investigation, adding a large sample of adolescents from Northern Russia to the research base on interpersonal provocation may enable future studies, such as meta-analytic investigations, that can compare Western and non-Western cultures. This study further clarifies how aggressive/interpersonal violent behavior can be shaped by traumatic experiences and supports the relevance of Western cognitive-emotional constructs in the context of trauma in a Russian sample, hence adding evidence to the universalistic scope of the social information processing model of aggression and emphasizing the need for research on macrolevel culture and societal factors. PTS symptoms may be important for the regulation of negative emotional experiences and might potentially be associated with different reactions to interpersonal provocation. This finding has direct implications for the rehabilitation of traumatized individuals, and should be taken into account when planning the treatment of adolescents with PTS (Kaczkurkin et al., 2016). Our results also highlight the importance of the gender-specific aspects of cognitive-emotional processing of trauma, which may serve as an important treatment target in adolescents with PTSD (Shein-Szydlo et al., 2016).
